# Peer Support Activities for Veterans, Serving Members, and Their Families: Results of a Scoping Review

**DOI:** 10.3390/ijerph20043628

**Published:** 2023-02-18

**Authors:** Jean-Michel Mercier, Fardous Hosseiny, Sara Rodrigues, Anthony Friio, Suzette Brémault-Phillips, Duncan M. Shields, Gabrielle Dupuis

**Affiliations:** 1 Atlas Institute for Veterans and Families, Ottawa, ON K1Z 7K4, Canada; 2 National Police Federation, Ottawa, ON K2P 1P1, Canada; 3 Heroes in Mind Advocacy and Research Consortium, Faculty of Rehabilitation Medicine, University of Alberta, Edmonton, AB T6G 2R3, Canada; 4 Faculty of Medicine, University of British Columbia, Vancouver, BC V6T 1Z4, Canada

**Keywords:** peer support, peer group, veterans, well-being, scoping review, social support, military personnel, military family

## Abstract

For many, including military veterans and their families, support between individuals with shared lived experiences, or peer support, has long been utilized as a way to support each other through many different challenges. Building on other reviews and guided by the seven domains of well-being in the Canadian veteran well-being framework, the objective of this paper is to describe and catalogue the nature of peer support activities and related outcomes in the veteran, serving member, and family member populations. A scoping review following the five stages outlined by Arksey and O’Malley was conducted; it was guided by the question: What is currently known about peer support activities for veterans, serving members, and their families that has been evaluated in the literature? In total, 101 publications from 6 different countries were included in this review and catalogued based on publication characteristics, participant information, peer support activity information, and peer information. Peer support activities have the potential to positively influence the well-being of veterans, serving members, and their families on a holistic level across multiple domains. This scoping review highlights the existing gaps in the literature and provides an important foundation for future research on peer support for these populations, specifically in the Canadian context.

## 1. Introduction

For many, including military veterans and their families, peer support has long been utilized as a way to support each other through many different challenges, health-related or otherwise [1,2]. While many definitions exist to describe peer support activities, the concept of shared life experiences between individuals remains constant [3,4,5]. Specific to the military and veteran field, though many peer support activities are community-based, peers are also being utilized more formally in the provision of programs and activities by government departments such as the Department of National Defence in Canada and the Veterans Health Administration in the United States [1,2,3,6,7,8]. Considering the increased interest in peer-supported activities in the literature, the question of the effectiveness of these activities on improving well-being is one that is becoming increasingly important to answer [2,9].

A clear definition of well-being and its components, however, currently seems to lack consensus [10,11,12,13,14]. Looking to address this need and to facilitate future research for Veterans Affairs Canada’s (VAC) programs and policies, efforts were undertaken by VAC to create a well-being framework specific to the Canadian veteran population and their families [10]. With the social determinants of health as a foundation, the final framework proposed that holistic well-being is made up of seven domains: employment and meaningful activity or purpose, finances, health, life skills and preparedness, social integration, housing and physical environment, and cultural and social environment [10,15,16]. A detailed description of this veteran well-being framework is outside the scope of this paper but can be found in Thompson et al.’s publication [10]. Measuring the indicators for each of these domains can serve as a way to tailor programs and services for families and veterans across their life course “from cradle to the grave” [10] (p. 4). The framework also highlights the importance of the military-civilian transition (MCT) period on well-being, which begins before the serving member’s release [10]. For this framework to be best utilized to improve well-being, the related activities and policies must align with the same components [10]. These activities include those utilizing peers, which are recognized in the aforementioned framework as a type of service that can contribute to overall well-being [10].

Currently, the peer support literature is quite heterogeneous, which may be contributing to the lack of empirical support on effectiveness [2,17]. Although previous reviews have explored the peer support literature, a gap still exists relating to the landscape of such activities on multiple, holistic dimensions of well-being for veterans, serving members, and their families. Specific to these populations, existing reviews such as those by Williams et al. [18] and Bird [19] focused on a specific activity category (one-to-one support), or a specific type of program (peer outdoor support therapy programs), respectively. Other reviews on peer support activities not specific to military populations include those related to particular conditions, such as mental illness or depression [4,17,20], substance use or addiction [21,22], and cancer [23,24]. Additionally, other publications focus on specific population groups, such as children with neurodevelopmental and intellectual disabilities [25], individuals with neurological conditions [26], and prisoners [27], among others.

Recognizing the increase in peer support activities for veterans, serving members and their families and the lack of empirical support for these activities, Ramchand et al. [2] attempted to bring clarity to the field by categorizing various peer-supported interventions’ components, peer roles, outcomes, and effectiveness [2]. Completed for the eventual benefit of veterans, serving members, and their families, Ramchand et al.’s [2] review considered randomized controlled trials for all populations. Building off this work and using it as a foundation, the current scoping review includes many types of study designs and publications. The current review aims to describe the nature of all evaluated peer support activities specific to veterans, serving members, and their families. Additionally, the current review aims to align presented outcomes within the aforementioned Canadian veterans’ well-being framework created by VAC. Peer support activities were catalogued according to their characteristics, population and peer characteristics, and by associated domains of well-being. Drawing on these results, this paper intends to fill a knowledge gap by presenting and clarifying the current state of the international peer support literature for veterans, serving members, and their families, and laying the foundation for future research, especially on the effectiveness of peer support on multiple dimensions of well-being in the Canadian veteran and military context.

## 2. Materials and Methods

The authors conducted a scoping review following the five stages outlined by Arksey and O’Malley, and expanded on by Levac, Colqunhoun, and O’Brien [28,29]. This type of review was deemed appropriate as the research on peer support is ever emerging and the authors were looking to “examine the extent, range and nature” of peer support activities [28] (p. 21).

### 2.1. Identification of Research Question

Keeping a broad approach to the search, the scoping review was guided by the question: what is currently known about peer support activities for veterans, serving members, and their families that has been evaluated in the literature? Considering the wide scope of the research question, the following sub-questions were developed to further refine the review:

- What are the characteristics of the veterans, serving members, and their families participating in these peer support activities?
- What are the types and characteristics of the peer support activities evaluated in the literature for veterans, serving members, and their families?
- Which domains of well-being are these activities aiming to improve?
- What are the gaps and limitations in the literature on peer support activities for veterans, serving members, and their families?

### 2.2. Identification of Relevant Publications

The search strategy was created in consultation with a research librarian and advisory group consisting of individuals with lived and living expertise, as well as professional and academic expertise. Six electronic databases were searched in July 2020: Medline, Embase, PsychInfo, Cochrane, CINAHL, and Web of Science. A search of other credible electronic sources outside of peer-reviewed journals was also completed in January 2021 in order to capture the full spectrum of publications. These included Google, Veterans Affairs Canada’s website, National Institute for Health and Clinical Excellence, Centre for Disease Control and Prevention, Department of National Defence Canada’s website, and RAND Corporation’s website. The searches were conducted with a combination of terms related to peer and social support in military, veteran, first responders or other public safety personnel (PSP), and family member populations. The search terms chosen were used to capture the broad extent of peer support activities, and serving members were added as a population of interest, considering the MCT period begins while individuals were still serving. The search was limited to English or French publications from January 2000 or later. Limitations due to language were added due to translation capacity, with the timeframe chosen to be large enough to capture the breadth of the relevant literature. No limitations were added in relation to the outcomes. The search was re-run across all sources and databases in December 2021 using the same strategy to update the results. The full search strategy is available in the Supplementary File S1, with an example from one database available in Table 1.

### 2.3. Article Selection

A team approach was utilized for the publication selection process [28]. Prior to initiating the screening, a sample of abstracts was reviewed by each reviewer to assess understanding of the inclusion and exclusion criteria. Two independent reviewers reviewed the abstracts, appraising each against the inclusion and exclusion criteria. Conflicts between reviewers were addressed through consensus, with third-party consultation by a third reviewer when required. Each reviewer then reviewed the full text publications, utilizing the same inclusion and exclusion criteria. To effectively answer the research questions while taking into account the available resources and the identified publications, post hoc inclusion and exclusion criteria were added [28,29]. The added criteria further limited the scope to include only primary studies describing a specific peer support activity with an evaluation component included. While initially part of the search, the publications relating solely to PSP were excluded to narrow the scope of the review, as were dissertations, studies relating solely to training programs for peer supporters, and editorials/commentaries. The article selection process was completed in Covidence, an online review workflow platform. To capture the breadth of the literature and clarify the publications to include, a clear definition of peer support activities had to be identified. As no current consensus exists, the definition of a peer support activity was determined by the authors with the support of the study’s advisory group as being any activity providing any kind of support from one or many individuals to another with a shared lived or living experience.

### 2.4. Data Collection

Using a data extraction form initially created in consultation with the study’s advisory group and guided by Cochrane’s data collection forms for qualitative and quantitative studies, two reviewers were responsible for the independent data extraction from the included articles. A third reviewer provided consensus on any data extraction conflicts. After an initial extraction of a sample of studies, the data extraction form was revised. The final data extraction was then completed using the revised form.

### 2.5. Collating and Summarizing Results

The final data, presented in the results section, was collated based on four categories:

1. Publication characteristics: including year of publication, country, journal, and design of the publication;
2. Participant information: including group (veteran, serving member, families), health condition, phase of the life course, and sex and gender;
3. Activity information: including name, format, modality, timing, duration and intensity, supervision, cost, reported adverse effects, and measured outcomes;
4. Peer information: including main role of peer, integration in a clinical team, training, and category related to remuneration. A ‘yes’ or ‘no’ approach to the training component was used, due to the varied nature of training described in the literature.

For the activity information category, all evaluated outcomes from publications were identified and categorized under the related domain of well-being. To identify if peer support activities were associated with an improvement in well-being, outcomes were also categorized as positively associated with the peer support activity, or not. The identification of positive association varied by study design. For controlled studies, only statistically significant (*p* < 0.05) between-group differences were included. For uncontrolled studies, only outcomes with a statistically significant (*p* < 0.05) improvement were included. Lastly, for qualitative studies, an outcome was included if the authors identified it as a theme commonly mentioned by participants. In the timing category, activities for which peer support was provided in real time were categorized as synchronous, and those for which it was not, such as recorded videos, were categorized as asynchronous.

Related to the peer information category, the definitions and roles described by Ramchand et al. [2], which built upon those proposed by Webel et al. [30], were used to categorize the role of the peers and were adapted when necessary. The initial roles vary from peer support, described as unstructured “buddy” support, up to the peer case manager, used to describe a peer with a service coordination and management role. Other roles included peer counsellor, for peers providing knowledge and guidance; peer educator, for peers providing education on a topic based on a curriculum; and peer facilitator, for peers “responsible for facilitating group interactions” [2] (p. 159). For this review, the roles remain as named by Ramchand et al. [2], with the exception of “peer support”, which was renamed to “informal peer support” by the authors, considering the definition of peer support activity used in this review as being all-encompassing of these types of roles. The term “peer supporter” is used in this review to describe every type of role.

## 3. Results

**Figure 1:**
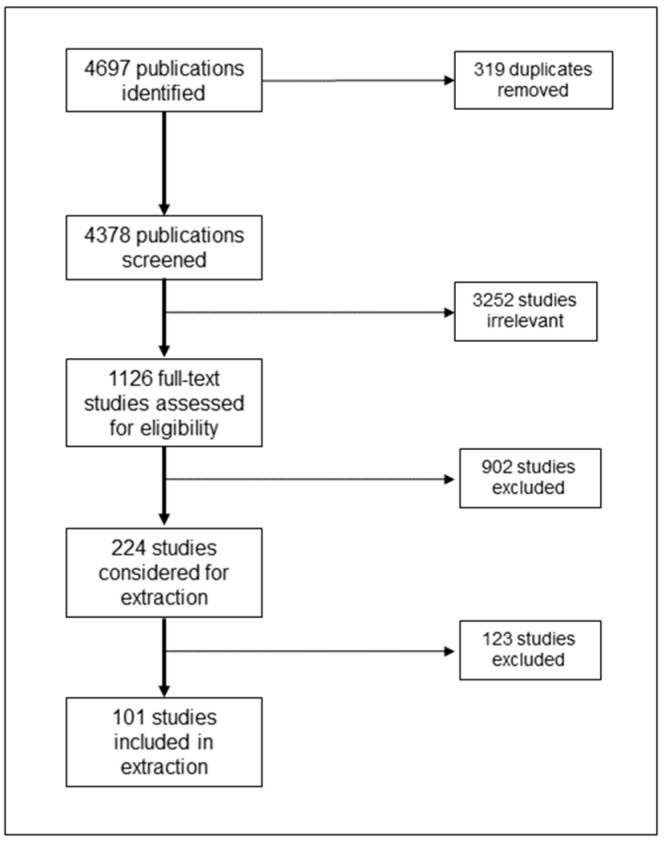
PRISMA diagram.

After the removal of duplicates, 4378 title/abstracts were screened. Of these, 3252 publications were excluded, leaving a total of 1126 to be reviewed. After full text review and application of the post hoc criteria, the team excluded another 1025 publications, providing a total of 101 publications meeting the inclusion criteria (Figure 1). Considering the purpose of the current scoping review, no critical appraisal of the evidence was conducted.

**Figure 2:**
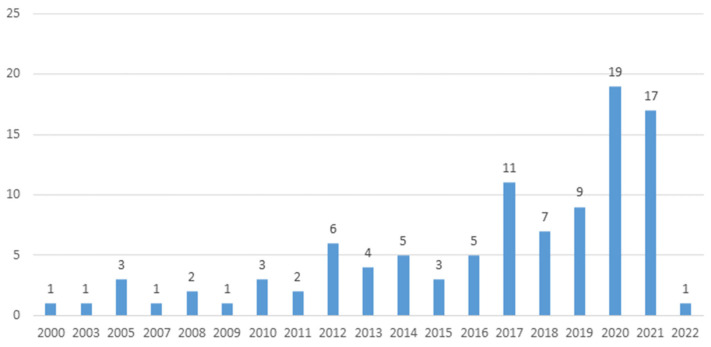
Count of studies by year of publication.

A total of 101 publications from 6 different countries were included in this review, with the majority coming from researchers in the United States (3 of these publications included a co-author from Spain; the participants from these studies were located in the United States) (n = 85; 84%). The publications also came from the United Kingdom (n = 6; 6%), Australia (n = 3; 3%), Iran (n = 3; 3%), Canada (n = 2; 2%), and Israel (n = 2; 2%). The details on the years of publication are available in Figure 2, with many of the included documents published either in 2020 (n = 19; 19%) or 2021 (n = 17; 17%).

Combinations of many different study designs were utilized in the included publications. The most common designs were experimental (n = 32; 32%), mixed-method (n = 22; 22%), quasi-experimental (n = 22; 22%), and qualitative (n = 16; 16%) (Table 2). Of all publications, 27 (27%) were identified as pilot studies, with most being uncontrolled (n = 20).

### 3.1. Participant Characteristics

Although the inclusion criteria for the search included peer support activities for veterans, serving members, and their families, most of the included publications reported on activities for veteran participants only (n = 77; 76%) (Table 3). Looking at sex and gender, the publications were categorized as having “almost all/all” participants of one sex or gender if over 80% were identified as that sex/gender, “majority” if between 60–80% of participants were of the same sex/gender, and “both” if the other categories did not apply. Only 11% of all reviewed publications (n = 11) included a majority or almost all/all female participants. All but one of these eleven were publications conducted with families, with three also including serving members, and one with only serving members. None of the publications with veteran populations were conducted with samples involving solely or predominantly female participants. Of note, none of the identified publications focused on peer support activities for veterans, families, or serving members who were part of the 2SLGBTQ+ community, individuals who have experienced military sexual trauma (MST), or those who identified as Indigenous. Only two publications focused specifically on people of color.

The majority of the publications reported on activities for participants with particular health conditions (n = 72; 71%). Specifically, 50% of all publications included participants with mental health conditions (n = 51) (Table 3).

### 3.2. Activity Characteristics

To be included in the analysis, the publications had to relate to specific peer support activities. Some of the included publications reported on the same activity, and the counts presented reflect those of individual publications, not of unique activities. Of the 101 publications, 59 unique activities were identified and 24 activities were not named (Table A1).

The publications were categorized based on the format of the evaluated activity, modality, timing, activity supervision, and measured outcomes. The publications evaluated activities that were mostly delivered in-person (n = 58; 57%), with the format for all activities almost evenly divided between one-to-one support (n = 45; 45%), and group-based support (n = 46; 46%).

The activities were also categorized based on the timing of the delivery. In total, 93 publications (92%) related to a peer support activity being delivered synchronously. All six publications evaluating asynchronous peer support were in relation to online or remotely delivered activities; however, not all publications reporting on activities delivered online/remotely were asynchronous (n = 4 synchronous).

Although separate, peer support activities can occur in conjunction with other care. Some publications used a peer support activity not as the main intervention but adjunctively to enhance the main intervention (n = 12). This included using peers to help reduce attrition with therapist-delivered prolonged exposure therapy and to support engagement with online programs and mobile applications. The complete results of all characteristics are available in Table 4 below.

### 3.3. Peer Characteristics

In terms of peer roles, relating to the aforementioned categories highlighted by Ramchand et al. [2], peer supporters most frequently acted as informal peer supporters (n = 34) and counsellors (n = 30). This was followed by facilitators (n = 20), educators (n = 14), and case managers (n = 3). In some instances, peer supporters were also included as part of the broader clinical team (n = 35). The peer supporters included in the clinical teams most frequently fulfilled the role of counsellors (n = 15); however, every other role was utilized as part of the clinical team at least once.

### 3.4. Veterans’ Well-Being Framework

The publications on peer support activities were further categorized based on the measured outcomes related to the domains of well-being and the phases of the life course. All measured outcomes from publications were categorized into the corresponding domain of well-being, with publications evaluating most frequently the association between the peer support activity and the outcomes related to the health domain (n = 56; 55%). Other outcomes evaluated included those related to life skills (n = 44; 44%), social integration (n = 39; 39%), purpose (n = 13; 13%), housing and physical environment (n = 4; 4%), and culture and social environment (n = 2; 2%). Twenty publications explored outcomes which could not be categorized under any of the domains of well-being, most of which were related to the program evaluation.

Once all measured outcomes were categorized within the related domain of well-being, those reported by the publications’ authors as being positively associated with the peer support activity were identified. The outcomes positively associated with a peer support activity were most frequently related to the health domain (n = 34) (Table 4). This translates to 61% of the 56 publications evaluating outcomes related to the health domain, showing a positive association between the activity and the outcomes. Among others, the outcomes associated with the health domain included mental health condition-related descriptors (e.g., PTSD or depression symptoms), glucose control, and weight loss. The publications also identified a positive association between peer support activities and outcomes related to the life skills (n = 30) and social integration (n = 26) domains. A positive association was reported in 68% of all publications related to the life skills domain, and 67% of publications related to the social integration domain reported a positive association. The outcomes associated with the life skills domain included measures of healthy daily habits, self-efficacy/confidence, coping skills, and others, while the indicators in the social integration domain included perceived social support and connectedness. Less frequently, some publications reported that the peer support activity was positively associated with the employment or other meaningful activity/purpose domain (n = 6), the housing and physical environment domain (n = 2), and the culture and social environment domain (n = 1). A positive association between the activity and the outcomes was reported in 46% of all publications evaluating employment or other meaningful activity/purpose-related outcomes, 50% of those evaluating housing and physical environment-related outcomes, and 50% of those evaluating culture and social environment-related outcomes.

**Figure 3:**
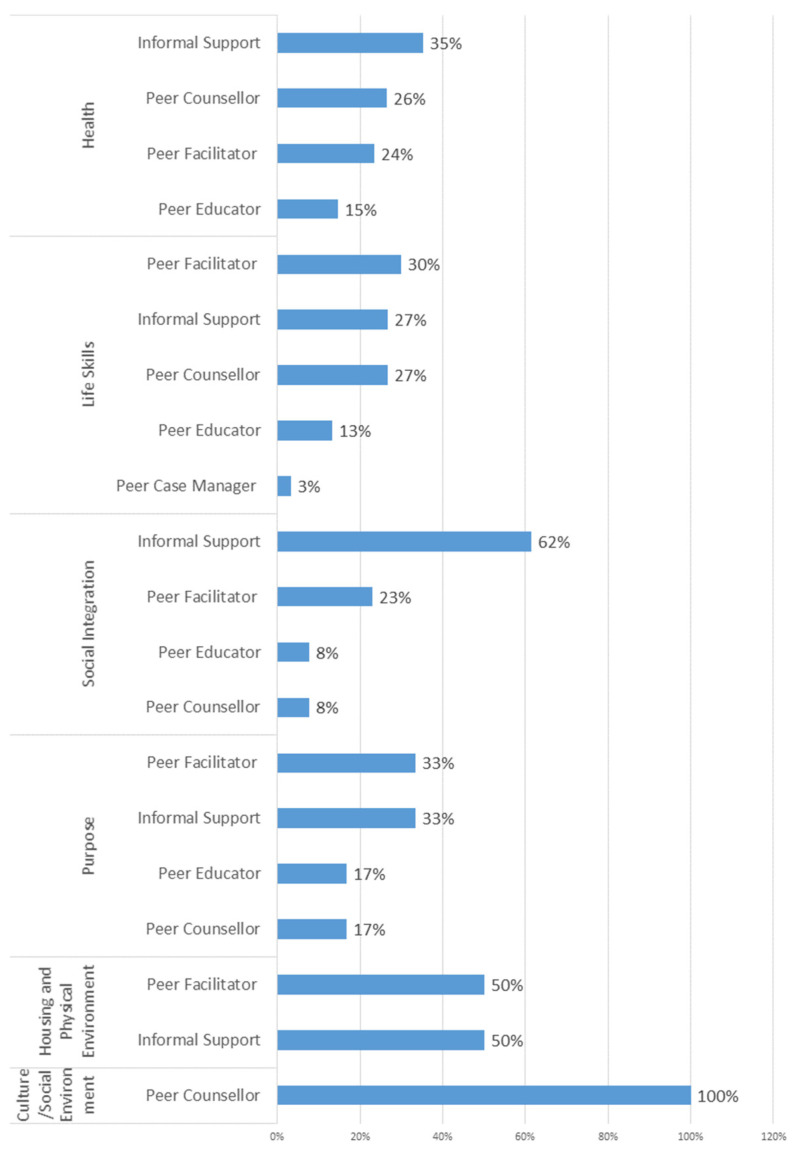
Main peer role by positively associated domain.

An attempt was made to categorize the publications according to the phase of the life course of the participants; however, the ability to do so was limited because the time since discharge was only rarely presented. Lastly, an analysis was conducted to identify the trends in the role of the peer supporter by associated well-being domains for positively associated outcomes. Considering each associated domain by publication, peer supporters were more frequently involved as informal support for both the health domain (35%) and the social integration domain (62%) when a positive association was identified (Figure 3). The details of the peer role and domains of well-being per publication are available in Table A2 in the Appendix A.

## 4. Discussion

This scoping review identified 101 relevant publications about peer support activities for veterans, serving members, and their families, demonstrating the breadth of peer support activities evaluated in these populations. With the vast majority of publications coming from the United States, it was not possible to compare activities between countries, therefore, all publications were grouped and analyzed together. Some authors have indicated a gradual increase in peer support publications from Europe and other countries, however, the findings from this review were unable to verify this trend in relation to veterans, serving members, and their families [31].

This review found that peer support activities in the literature are evaluated using various designs, many based on pilot studies. The presence of many pilot studies should be considered when interpreting the results of this review, as findings from these types of publications could differ with larger sample sizes. The increase in the number of publications from the last two years, as well as the presence of many pilot studies, emphasizes the emergent nature of the literature in this area and the increased attention that peer support activities are receiving in the veteran, serving member, and family member populations.

### 4.1. Participant and Activity Characteristics

The wide scope of this review allowed the authors to identify the myriad of components currently being utilized and evaluated in the delivery of support from peers to veterans, serving members, and their families. While the majority of identified publications evaluated synchronously delivered programs for male veterans with a mental health condition, a vast heterogeneity was present in relation to the other catalogued characteristics of activities and participants. Additionally, the successful categorization of peer roles using types of peers described by Ramchand et al. [2] supports this role characterization method for future studies aiming to evaluate peer support activities in the veteran, serving member, and families populations. Although outside of the scope of this current review, future publications should attempt to identify the existence of associations between activity characteristics, peer supporter roles, and associated outcomes and domains. Identifying these associations could help tailor peer support activities according to individual situations and needs.

### 4.2. Domains of Well-Being

Recognizing the limitations in the current method of identifying positive associations between measured activity and well-being, a strength of this review is the categorization of outcomes by well-being domain. Although some publications did not report an improvement, this study found that positive associations between peer support activities and six of the seven domains, excluding finances, were identified by at least one publication. Considering this, it can be concluded that peer support may have the potential to positively influence the well-being of veterans, serving members, and their families holistically.

Categorizing the evaluated outcomes by domain of well-being also revealed that the main interest of evaluated peer support activities lies in three of the seven domains. In the identified publications, the outcomes related to health, life skills, and social integration were more frequently mentioned or evaluated. On the other hand, the outcomes related to purpose, housing and physical environment, and culture and social environment were only rarely included, with no identified studies including measures categorized to the finance domain. Although more research evaluating peer support activities and their association to improving all domains of well-being is needed, it would be important for future research to consider including measurements for the latter four domains, as well-being is considered by VAC as being made up of all seven domains [10]. A true picture of the potential for peer support activities for veterans, serving members, and their families may only be achieved if future research takes into account all the domains when evaluating these activities.

Although evaluation of the alignment between the well-being framework and published peer support activities was not an objective of the current review, it was found that 80% of publications (n = 81) evaluated at least one outcome that aligned with a domain presented in the framework. This categorization was meant to test this method for future peer support studies in this population, while aligning with the recommendations of select authors suggesting the need to look beyond measures of clinical recovery to better assess the effectiveness of peer support activities [32].

### 4.3. Gaps and Limitations in the Literature

The findings from this review allowed the authors to identify gaps in the current literature. First, only two Canadian publications were identified, limiting the generalizability of the results within the Canadian context. Furthermore, only a few, or no, publications were found to be specifically evaluating peer support activities for serving members or family members, for individuals who have experienced military sexual trauma (MST), in 2S/LGBTQ+ populations, in Indigenous populations, or in women veterans. Considering the heterogeneous nature of Canadian serving members and veterans, unique sex- and gender based-differences, and recent developments with regards to individuals who have experienced MST and the LGBT Purge in the Canadian Armed Forces, future research should be conducted to evaluate the use of peer support activities for a more representative spectrum of veterans, serving members, and their families [33,34,35,36,37].

Finally, the included publications rarely considered the time since discharge when evaluating the peer support activities for veterans. Considering the differing nature of needs and well-being during different phases of the life course, identifying and reporting time since discharge could prove useful in expanding knowledge on peer support activities and their outcomes, and should be considered in future research [10].

### 4.4. Study Limitations

Though this scoping review can be used as a foundation for future research in the area of peer support for Canadian veterans, serving members, and their families, some limitations should be considered when interpreting the results beyond the ones already mentioned. The first relates to the descriptive nature of the analysis. Based on the purpose and nature of this review, no critical appraisal of individual sources was conducted and many study designs were considered. This limits the authors’ ability to confirm the benefits of the peer support activities on each domain of well-being and future research should aim to reach more definitive conclusions by establishing the strength of the available evidence, considering research designs and effect sizes. Another limitation is related to the categorizations of outcomes to their respective well-being domain. The well-being framework used is composed of seven domains, however, not all of them are mutually exclusive and some overlap may exist between domains. For this study, the authors categorized outcomes based on subjective best fit, which may have led to misclassification. A final limitation is related to the search criteria used. Although the search for publications was kept as wide as possible, some relevant articles may have been missed due to search terms used.

## 5. Conclusions

The purpose of this review was to describe the nature of peer support activities specific to veterans, serving members, and their families and to align their outcomes within VAC’s Canadian veterans’ well-being framework. Already utilized by some government departments, peer support activities in these populations are receiving increased attention in the literature and have the potential to improve well-being across all domains. This scoping review provides an important foundation for future research related to peer support in Canadian veterans, serving members, and their families. Utilizing a consultative approach with an advisory group of varied expertise, this review builds on existing research and is another step towards standardizing the peer support vocabulary, as well as the way these activities are designed, evaluated, and presented in the Canadian context.

## Figures and Tables

**Table 1:** Embase search strategy.

Search Strategy	Results
1 peer group/	26,856
2 (peer* adj3 (support or group* or mentor* or lead* or coach* or counsel* or service* or specialist* or outreach or network* or volunteer* or education*)).tw.	22,342
3 (peer led or peer based or peer deliver*).tw.	2410
4 *community care/	23,893
5 (buddy or buddies).tw.	1309
6 exp support group/	13,483
7 support group*.tw.	12,509
8 (mutual support or mutual help).tw.	1365
9 (para-professional* or paraprofessional*).tw.	1293
10 (community adj2 network*).tw.	2943
11 or/1–10	88,081
12 veteran/	30,660
13 exp military personnel/ or military service/ or military spouse/ or military family/	8075
14 rescue personnel/	8437
15 police/	14,515
16 fire fighter/	3569
17 (Military or paramilitary or armed force* or veteran* or armed service* or servicewomen or servicemen or air-personnel or defense force* or defence force* or service personnel or navy or air force or sailor* or soldier* or infantryman or Civil-defense or Troops or coast guard or submariner* or active duty or enlisted personnel or reserve personnel or police officer* or RCMP or firefighter* or firem* or EMT or EMTs or EMS or (Emergency Medical adj2 (personnel or technician*)) or paramedic* or (public safety adj3 (professional* or official* or personnel*)) or “first responder*” or “search and rescue” or ambulance or ((law enforcement or corrections or correctional) adj (worker* or staff or personnel or officer*)) or security guard* or security personnel or sheriff* or (border adj3 (agent* or personnel or security)) or emergency manager* or ((“911” or emergency) adj3 (dispatcher or personnel))).tw.	231,843
18 12 or 13 or 14 or 15 or 16 or 17	251,323
19 11 and 18	1455
20 animals/ not humans/	1,405,292
21 19 not 20	1455
22 limit 21 to yr=“2000 -Current”	1307

* denotes wildcard.

**Table 2:** Information of included publications.

Variable	Number of Publications (%)
Country of Publication	
United States	85 (84.2)
UK	6 (5.9)
Iran	3 (3.0)
Australia	3 (3.0)
Canada	2 (2.0)
Israel	2 (2.0)
Design	
Experimental	32 (31.7)
Mixed-methods	22 (21.8)
Quasi-experimental	22 (21.8)
Qualitative	16 (15.8)
Observational	5 (5.0)
Other Publication	2 (2.0)
Case Study	2 (2.0)

**Table 3:** Information of participants in included publications.

Variable	Number of Publications (%)
Main Population	
Veterans	77 (76.2)
Combination	10 (9.9)
Families	9 (8.9)
Serving members	5 (5.0)
Health Condition	
No condition specified	29 (28.7)
Metabolic/Cardiovascular	15 (14.9)
PTSD	15 (14.9)
Non-specified mental health	11 (10.9)
Other mental health	8 (7.9)
Dual diagnosis	7 (6.9)
Substance use disorder	4 (4.0)
Chronic pain	4 (4.0)
Depression	4 (4.0)
Other condition	3 (3.0)
Cancer	1 (1.0)
Sex and Gender	
Almost all or all male	67 (66.3)
Majority male	11 (10.9)
Almost all or all female	9 (8.9)
Not reported	7 (6.9)
Both	5 (5.0)
Majority female	2 (2.0)

**Table 4:** Information of peer support activities and peer characteristics in included publications.

Variable	Number of Publications (%)
Format	
Group	46 (45.5)
One-to-one	45 (44.6)
Combination	8 (7.9)
Not reported	2 (2.0)
Modality	
In-person	58 (57.4)
Choice	11 (10.9)
Phone	10 (9.9)
Online/Remotely	10 (9.9)
Combination	10 (9.9)
Not reported	2 (2.0)
Timing	
Synchronous	93 (92.1)
Asynchronous	6 (5.9)
Combination	2 (2.0)
Peer Role	
Informal peer support	34 (33.7)
Peer counsellor	30 (29.7)
Peer facilitator	20 (19.8)
Peer educator	14 (13.9)
Peer case manager	3 (3.0)
Peer Supervision	
Yes	83 (82.2)
No mention	18 (17.8)
Peer Part of Clinical Team	
No	65 (64.4)
Yes	35 (34.7)
N/A	1 (1.0)
Peer Training	
Yes	73 (72.3)
No	25 (24.8)
Not reported	3 (3.0)
Evaluated Domains ^1^	
Health	56 (55.4)
Life Skills	44 (43.6)
Social integration	39 (38.6)
N/A	20 (19.8)
Employment and meaningful activity/purpose	13 (12.9)
Housing and physical environment	4 (4.0)
Culture/social environment	2 (2.0)
Positively Associated Domains ^1^	
N/A	44 (43.6)
Health	34 (33.7)
Life skills	30 (29.7)
Social integration	26 (25.7)
Employment and meaningful activity/purpose	6 (5.9)
Housing and physical environment	2 (2.0)
Culture/social environment	1 (1.0)

^1^ Publications could be associated with multiple domains.

## Data Availability

For data supporting the reported results, please contact the authors of this review.

## Supplementary Materials

The following supporting information can be downloaded at: https://www.mdpi.com/article/10.3390/ijerph20043628/s1, File S1: Full search strategy.

## Appendix A

**Table A1:** Counts of unique peer support activities.

Activity Name	Number of Publications
Not Named	24
Vet-to-Vet	5
Trauma Risk Management (TRiM)	3
Evaluation of a Peer Coach-Led Intervention to Improve Pain Symptoms (ECLIPSE)	2
Improving Pain using Peer-Reinforced Self-Management Strategies (IMPPRESS)	2
Group-intensive peer support (GIPS) model for the Housing and Urban Development-Veterans Affairs Supportive Housing (HUD-VASH) program	2
Patient Aligned Care Team (PACT); Homeless-oriented PACT (H-PACT)	2
Taking Charge of My Life and Health (TCMLH)	2
Posts Working for Veterans’ Health (POWER)	2
MOVE!+UP	2
AMPS (Administering MISSION Vet using Peer Support)	2
Mentors Offering Maternal Support (MOMS)	2
Peer Supported Beating the Blues (PS-cCBT)	2
Homefront	2
The VA Palo Alto Health Care System (VAPAHCS) Peer Support Program (PSP)	2
Stand Down: Think Before You Drink + Peer Support	1
The Military Spouse Online Autism Relocation Readiness (MilSOARR)	1
Quick Reaction Force	1
Trojan’s Trek (TT) Peer Outdoor Support Therapy (POST)	1
Living-Well	1
Buddy-Care	1
Mentorship for Addictions Problems to Enhance Engagement to Treatment (MAP-Engage)	1
Veteran Coffee Socials	1
Mission Strong Booster Session	1
Shared Medical Appointments	1
Motivational Coaching to Enhance Mental Health Engagement in Rural Veterans (COACH)	1
The Artful Grief Studio	1
MOVE OUT	1
The Stanford Program (chronic condition self-management [CCSM])	1
Care Coordination Home Telehealth (CCHT)	1
Thinking Forward with Peer Support	1
VA CONNECT	1
Next Mission; Women Warriors	1
Big Brother Program	1
Caring Cards	1
Reciprocal Diabetes Peer Support Program (RPS) with Nurse Care Management (NCM)	1
Operational Stress Injury Social Support (OSISS) Peer Support Network (PSN)	1
Spark People (SP)	1
Outdoor Recreational activities	1
Armed Forces and Veterans’ Breakfast Clubs (AFVBCs)	1
Depression Intervention, Actively Learning and Understanding With Peers (DIAL-UP)	1
The Exposure Therapy Peer Support Program	1
Peer Enhanced Exposure Therapy (PEET)	1
The Right Turn	1
The Strong Military Families (SMF) intervention	1
Vets & Friends	1
The Wellness Program	1
Empowering Patients in Chronic Care	1
AboutFace	1
Understanding Suicide	1
Post War: Survive to Thrive Program	1
VA Student Partnership for Rural Veterans (VSP)	1
Adapted Maintaining Independence and Sobriety through Systems Integration, Outreach, and Networking-Veterans Edition (MISSION-Vet)	1
Veteran Supported Education Treatment Manual (VetSEd)	1
Proactive, Recovery-Oriented Treatment Navigation to Engage Racially Diverse Veterans in Mental Healthcare (PARTNER-MH)	1
Project The Outreach and Rehabilitation Center for Homeless Veterans (TORCH) Peer Mentor Program	1
VETS PREVAIL	1
Peer-delivered Whole Health Coaching	1
Web MOVE	1
Peers Enhancing Recovery (PEER)	1

**Table A2:** Detailed evidence table of all publications.

Citation	Population Group	Peer Role	Measured Domain	Positively Associated Domain
Hall et al. 2020 [38]	Veterans	Peer Counsellor	Life Skills, Health	Health
McDermott 2020 [39]	Veterans	Informal Support	Social Integration	Social Integration
Thoits et al. 2000 [40]	Veterans	Informal Support	Health, Social Integration	N/A
Geron et al. 2003 [41]	Families	Informal Support	Social Integration, Life Skills	Social Integration
Resnick and Rosenheck 2010 [42]	Veterans	Peer Facilitator	N/A	N/A
Perlman et al. 2010 [43]	Veterans	Informal Support	Health, Social Integration, Life Skills	Health, Social Integration, Life Skills
Heisler and Piette 2005 [44]	Veterans	Peer Counsellor	Health, Purpose, Life Skills	Purpose, Life Skills
Weissman et al. 2005 [45]	Veterans	Peer Facilitator	Housing and Physical Environment, Purpose, Social Integration, Health	Housing and Physical Environment, Purpose
Vakharia et al. 2007 [46]	Veterans	Informal Support	Life Skills, Health	Life skills, Health
Barber et al. 2008 [47]	Veterans	Peer Facilitator	Purpose	N/A
Resnick and Rosenheck 2008 [48]	Veterans	Peer Facilitator	Life Skills, Health	Life Skills, Health
Greenberg et al. 2011 [49]	Serving Members	Peer Educator	N/A	N/A
Tracy et al. 2011 [50]	Veterans	Peer Counsellor	Life Skills	N/A
Long et al. 2012 [51]	Veterans	Peer Counsellor	Health	Health
Greenberg et al. 2010 [52]	Serving Members	Peer Educator	Health, Culture/Social Environment	N/A
Heisler et al. 2010 [53]	Veterans	Informal Support	Health, Social Integration, Life Skills	Health, Social Integration
Eisen et al. 2012 [54]	Veterans	Peer Facilitator	Purpose, Social Integration, Health	N/A
Beattie et al. 2013 [55]	Mix: Veterans and Families	Informal Support	Health, Purpose, Life Skills	Health, Life Skills
Gabrielian et al. 2013 [56]	Veterans	Peer Educator	N/A	N/A
Mosack et al. 2013 [57]	Veterans	Peer Educator	Health, Life Skills, Social Integration	Health, Life Skills
Nichols et al. 2013 [58]	Families	Informal Support	Health, Life Skills, Social Integration	Health, Social Integration
Beehler et al. 2014 [59]	Veterans	Peer Facilitator	Life Skills, Social Integration	Life Skills
Mosack et al. 2012 [60]	Veterans	Peer Educator	Life Skills, Health	Health, Life Skills
Tsai and Rosenheck 2012 [61]	Veterans	Informal Support	Housing and Physical Environment, Health, Social Integration	Social Integration, Health, Housing and Physical Environment
VanVoorhees et al. 2012 [62]	Veterans	Peer Counsellor	Health, Life Skills, Culture/Social Environment	Health, Culture/Social Environment
Weis and Ryan 2012 [63]	Families, Serving Members	Peer Facilitator	Life Skills, Social Integration	N/A
Holtz et al. 2014 [64]	Veterans	Informal Support	Health	N/A
Matthias et al. 2020 [65]	Veterans	Peer Counsellor	Health, Life Skills	N/A
Tsai et al. 2014 [66]	Veterans	Informal Support	N/A	N/A
Whittle et al. 2014 [67]	Veterans	Peer Educator	Health, Life Skills	N/A
Bird 2015 [68]	Mix: Veterans and Serving Members	Peer Facilitator	Health, Purpose, Social Integration, Life Skills	Health, Purpose, Social Integration, Life Skills
Chinman et al. 2015 [3]	Veterans	Peer Case Manager	Purpose, Life Skills, Social Integration, Health	Life Skills
Matthias et al. 2015 [69]	Veterans	Peer Counsellor	Health, Life Skills, Social Integration	N/A
Valenstein et al. 2016 [70]	Veterans	Informal Support	Health, Purpose	N/A
Nelson et al. 2014 [71]	Veterans	Peer Counsellor	Health	Health
Cohen et al. 2017 [72]	Veterans	Informal Support	N/A	N/A
Fletcher et al. 2017 [73]	Veterans	Peer Educator	Social Integration	Social Integration
Vagharseyyedin et al. 2017 [74]	Families	Peer Facilitator	Social Integration, Purpose	Social Integration
Weis et al. 2017 [75]	Families, Serving Members	Peer Facilitator	Health, Social Integration, Life Skills	Health
Yoon et al. 2017 [76]	Veterans	Peer Counsellor	N/A	N/A
Young et al. 2017 [77]	Veterans	Peer Counsellor	Health	Health
Chinman et al. 2018 [78]	Veterans	Peer Educator	Health, Social Integration	N/A
Cheney et al. 2016 [79]	Veterans	Peer Counsellor	N/A	N/A
Ellison et al. 2016 [80]	Veterans	Peer Counsellor	Health, Life Skills	N/A
Jain et al. 2016 [81]	Veterans	Informal Support	Health, Social Integration	N/A
Matthias et al. 2016 [82]	Veterans	Peer Counsellor	N/A	N/A
Ellison et al. 2018 [83]	Veterans	Peer Counsellor	Health, Life Skills	Life Skills
Goetter et al. 2018 [84]	Veterans	Peer Case Manager	Life Skills	N/A
Gorman et al. 2018 [85]	Veterans	Peer Facilitator	Social Integration	Social Integration
Julian et al. 2018 [86]	Mix: Serving Members and Families	Informal Support	Life Skills	Life Skills
Messinger et al. 2018 [87]	Veterans	Informal Support	Health, Life Skills	N/A
Hernandez-Tejada et al. 2017 [88]	Veterans	Informal Support	N/A	N/A
Hernandez-Tejada et al. 2017 [89]	Veterans	Informal Support	N/A	N/A
Jones et al. 2017 [90]	Serving Members	Peer Counsellor	Health	Health
Resnik et al. 2017 [91]	Veterans	Peer Counsellor	N/A	N/A
Vagharseyyedin et al. 2018 [92]	Families	Peer Facilitator	Health	Health
Hamblen et al. 2019 [93]	Veterans	Informal Support	Health	N/A
Haselden et al. 2019 [94]	Families	Peer Facilitator	Health, Social Integration, Life Skills	Health, Social Integration, Life Skills
Kumar et al. 2019 [95]	Veterans	Peer Facilitator	Life Skills, Social Integration	Life Skills, Social Integration
Lott et al. 2019 [96]	Veterans	Peer Counsellor	Life Skills, Social Integration, Health	Life Skills, Social Integration, Health
McCarthy et al. 2019 [97]	Veterans	Peer Counsellor	Social Integration	N/A
Possemato et al. 2019 [98]	Veterans	Peer Educator	Health, Purpose	N/A
Romaniuk et al. 2019 [99]	Veterans	Peer Educator	Purpose, Health	Purpose, Health
VanVoorhees et al. 2019 [100]	Veterans	Peer Counsellor	N/A	N/A
Arney et al. 2020 [101]	Veterans	Informal Support	Social Integration, Life Skills, Health	Social Integration, Life Skills, Health
Azevedo et al. 2020 [102]	Veterans	Peer Facilitator	Social Integration, Life Skills	Social Integration, Life Skills
Blonigen et al. 2020 [103]	Veterans	Peer Counsellor	Life Skills, Health	Life Skills
Boehm et al. 2020 [104]	Mix: Veterans and Families	Informal Support	Social Integration	Social Integration
Eliacin et al. 2020 [105]	Veterans	Peer Counsellor	N/A	N/A
Ellison et al. 2020 [106]	Veterans	Peer Counsellor	Health, Housing	N/A
Harris et al. 2020 [107]	Veterans	Peer Educator	Life Skills	Life Skills
Hoerster et al. 2020 [108]	Veterans	Peer Facilitator	Health, Life Skills	Health, Life Skills
Matthias et al. 2020 [109]	Veterans	Peer Counsellor	Health	N/A
Pfeiffer et al. 2020 [110]	Veterans	Peer Counsellor	Health, Life Skills	Health, Life Skills
Johnson et al. 2021 [111]	Veterans	Peer Counsellor	Life Skills	Life Skills
van Reekum and Watt 2019 [112]	Veterans	Informal Support	Health, Social Integration	Health, Social Integration
Albertson et al. 2017 [113]	Veterans	Informal Support	Purpose, Health	Purpose, Health
DND 2005 [8]	Mix: Veterans and Serving Members	Peer Case Manager	N/A	N/A
Yeshua-Katz 2021 [114]	Mix: Veterans and Families	Informal Support	N/A	N/A
Turner et al. 2021 [115]	Veterans	Peer Counsellor	Health, Life Skills	Health, Life Skills
Strouse et al. 2021 [116]	Families	Informal Support	Social Integration, Purpose	Social Integration, Purpose
Seal et al. 2021 [117]	Veterans	Peer Educator	Health, Social Integration	Health, Social Integration
Schutt et al. 2021 [118]	Veterans	Peer Counsellor	Housing	N/A
Robustelli et al. 2022 [119]	Veterans	Peer Counsellor	N/A	N/A
Rajai et al. 2021 [120]	Families	Informal Support	Life Skills, Social integration	Life Skills, Social integration
Muralidharan et al. 2021 [121]	Veterans	Peer Facilitator	N/A	N/A
Gromatsky et al. 2021 [122]	Veterans	Informal Support	Health, Social Integration	Health, Social Integration
Kremkow and Finke 2022 [123]	Families	Peer Counsellor	Social Integration, Life Skills	Social Integration, Life Skills
Hernandez-Tejada et al. 2021 [124]	Veterans	Informal Support	N/A	N/A
Gebhardt et al. 2021 [125]	Veterans	Informal Support	N/A	N/A
Ehret et al. 2021 [126]	Mix: Veterans and Serving Members	Informal Support	Social Integration	Social Integration
Heisler et al. 2021 [127]	Veterans	Peer Facilitator	Health	Health
Coughlin et al. 2021 [128]	Serving Members	Peer Educator	N/A	N/A
Balmer et al. 2020 [129]	Mix: Veterans and Families	Informal Support	Social Integration, Life Skills	Social Integration, Life Skills
Abadi et al. 2021 [130]	Veterans	Peer Facilitator	Health, Life Skills	Health, Life Skills
Wheeler et al. 2020 [131]	Veterans	Informal Support	Health, Social Integration	Health, Social Integration
Villaruz Fisak et al. 2020 [132]	Serving Members	Informal Support	Health, Social Integration	N/A
Norman et al. 2020 [133]	Veterans	Informal Support	Social Integration, Life Skills, Health	Social Integration, Life Skills, Health
Haselden et al. 2020 [134]	Families	Peer Facilitator	Life Skills	Life Skills
Long et al. 2020 [135]	Veterans	Peer Counsellor	Health	N/A
Abadi et al. 2021 [136]	Veterans	Peer Educator	Health, Life Skills	Health, Life Skills
