# Supplementary material for: Peer Support Activities for Veterans, Serving Members, and Their Families: Results of a Scoping Review
Source: Int J Environ Res Public Health. 2023 Feb 18;20(4):3628. doi: 10.3390/ijerph20043628 (PMC9964749; doi:10.3390/ijerph20043628)
Supplement: Supplementary file 1 [file ijerph-20-03628-s001.zip › ijerph-2186308-supplementary.pdf]

Supplemental File S1: Full Search Strategy

Database: Ovid MEDLINE(R) ALL <1946 to December 06, 2021> Search Strategy:

----

1 Peer Group/ or Social Support/ (95262)
2 (peer* adj3 (support or group* or mentor* or lead* or coach* or counsel* or service* or specialist* or outreach or network* or volunteer* or education*)).tw. (16818)
3 (peer* and (support or group* or mentor* or lead* or coach* or counsel* or service* or specialist* or outreach or network* or volunteer* or education*)).kw. (589)
4 (peer led or peer based or peer deliver*).tw,kw. (1886)
5 Community Networks/ (7156)
6 (community adj2 network*).tw. (2349)
7 (buddy or buddies).tw,kw. (833)
8 Self-Help Groups/ or support group*.tw,kw. (15318)
9 (mutual support or mutual help).tw,kw. (1064)
10 (para-professional* or paraprofessional*).tw,kw. (1071)
11 or/1-10 (127934)
12 Veterans Health/ or Veterans/ (20321)
13 Military Personnel/ (42265)
14 exp Emergency Responders/ (13992)
15 (Military or paramilitary or armed force* or veteran* or armed service* or servicewomen or servicemen or air-personnel or defense force* or defence force* or service personnel or army or navy or air force or sailor* or soldier* or infantryman or Civil-defense or Troops or coast guard or submariner* or active duty or enlisted personnel or reserve personnel or police officer* or RCMP or firefighter* or firem* or EMT or EMTs or EMS or (Emergency Medical adj2 (personnel or technician*)) or paramedic* or (public safety adj3 (professional* or official* or personnel*)) or "first responder*" or "search and rescue" or ambulance or ((law enforcement or corrections or correctional) adj (worker* or staff or personnel or officer*)) or security guard* or security personnel or sheriff* or (border adj3 (agent* or personnel or security)) or emergency manager* or (("911" or emergency) adj3 (dispatcher or personnel))).tw,kf. (189372)
16 or/12-15 (207759)
17 11 and 16 (2275)
18 limit 17 to yr="2000 -Current" (1923)

Database: Embase Classic+Embase <1947 to 2021 December 06> Search Strategy:

----

1 peer group/ (26856)
2 (peer* adj3 (support or group* or mentor* or lead* or coach* or counsel* or service* or specialist* or outreach or network* or volunteer* or education*)).tw. (22342)
3 (peer led or peer based or peer deliver*).tw. (2410)
4 *community care/ (23893)
5 (buddy or buddies).tw. (1309)
6 exp support group/ (13483)
7 support group*.tw. (12509)
8 (mutual support or mutual help).tw. (1365)
9 (para-professional* or paraprofessional*).tw. (1293)
10 (community adj2 network*).tw. (2943)

11 or/1-10 (88081)
12 veteran/ (30660)
13 exp military personnel/ or military service/ or military spouse/ or military family/ (8075)
14 rescue personnel/ (8437)
15 police/ (14515)
16 fire fighter/ (3569)
17 (Military or paramilitary or armed force* or veteran* or armed service* or servicewomen or servicemen or air-personnel or defense force* or defence force* or service personnel or navy or air force or sailor* or soldier* or infantryman or Civil-defense or Troops or coast guard or submariner* or active duty or enlisted personnel or reserve personnel or police officer* or RCMP or firefighter* or firem* or EMT or EMTs or EMS or (Emergency Medical adj2 (personnel or technician*)) or paramedic* or (public safety adj3 (professional* or official* or personnel*)) or "first responder*" or "search and rescue" or ambulance or ((law enforcement or corrections or correctional) adj (worker* or staff or personnel or officer*)) or security guard* or security personnel or sheriff* or (border adj3 (agent* or personnel or security)) or emergency manager* or (("911" or emergency) adj3 (dispatcher or personnel))).tw. (231843)
18 12 or 13 or 14 or 15 or 16 or 17 (251323)
19 11 and 18 (1455)
20 animals/ not humans/ (1405292)
21 19 not 20 (1455)
22 limit 21 to yr="2000 -Current" (1307)

Database: APA PsycInfo <1806 to November Week 5 2021> Search Strategy:

----

1 Peers/ or Peer Relations/ (30573)
2 (peer* adj3 (support or group* or mentor* or lead* or coach* or counsel* or service* or specialist* or outreach or network* or volunteer* or education*)).tw. (22428)
3 (peer led or peer based or peer deliver*).tw. (1585)
4 (buddy or buddies).tw. (599)
5 support groups/ or social networks/ or support group*.tw. (23102)
6 (mutual support or mutual help).tw. (1450)
7 paraprofessional personnel/ or (para-professional* or paraprofessional*).tw. (3252)
8 Social Support/ (39645)
9 or/1-8 (106058)
10 Military Veterans/ (14919)
11 Military Personnel/ or Enlisted Military Personnel/ or Military Families/ or Military Deployment/ (13300)
12 first responders/ or exp emergency personnel/ (12673)
13 (Military or paramilitary or armed force* or veteran* or armed service* or servicewomen or servicemen or air-personnel or defense force* or defence force* or service personnel or navy or air force or sailor* or soldier* or infantryman or Civil-defense or Troops or coast guard or submariner* or active duty or enlisted personnel or reserve personnel or police officer* or RCMP or firefighter* or firem* or EMT or EMTs or EMS or (Emergency Medical adj2 (personnel or technician*)) or paramedic* or (public safety adj3 (professional* or official* or personnel*)) or "first responder*" or "search and rescue" or ambulance or ((law enforcement or corrections or correctional) adj (worker* or staff or personnel or officer*)) or security guard* or security personnel or sheriff* or (border adj3 (agent* or personnel or security)) or emergency manager* or (("911" or emergency) adj3 (dispatcher or personnel))).tw. (72885)
14 10 or 11 or 12 or 13 (79871)

15 9 and 14 (2106)
16 limit 15 to yr="2000 -Current" (1636)
17 limit 16 to ("0200 book" or "0240 authored book" or "0280 edited book") (154)
18 16 not 17 (1482)

Database: EBM Reviews - Cochrane Central Register of Controlled Trials <November 2021> Search Strategy:
----
1 Peer Group/ or Social Support/ (4677)
2 (peer* adj3 (support or group* or mentor* or lead* or coach* or counsel* or service* or specialist* or outreach or network* or volunteer* or education*)).tw. (4235)
3 (peer led or peer based or peer deliver*).tw. (882)
4 Community Networks/ (176)
5 (community adj2 network*).tw. (225)
6 (buddy or buddies).tw. (251)
7 Self-Help Groups/ or support group*.tw. (2437)
8 (mutual support or mutual help).tw. (135)
9 (para-professional* or paraprofessional*).tw. (244)
10 or/1-9 (11032)
11 Veterans Health/ or Veterans/ (1152)
12 Military Personnel/ (1040)
13 exp Emergency Responders/ (332)
14 (Military or paramilitary or armed force* or veteran* or armed service* or servicewomen or servicemen or air-personnel or defense force* or defence force* or service personnel or army or navy or air force or sailor* or soldier* or infantryman or Civil-defense or Troops or coast guard or submariner* or active duty or enlisted personnel or reserve personnel or police officer* or RCMP or firefighter* or firem* or EMT or EMTs or EMS or (Emergency Medical adj2 (personnel or technician*)) or paramedic* or (public safety adj3 (professional* or official* or personnel*)) or "first responder*" or "search and rescue" or ambulance or ((law enforcement or corrections or correctional) adj (worker* or staff or personnel or officer*)) or security guard* or security personnel or sheriff* or (border adj3 (agent* or personnel or security)) or emergency manager* or (("911" or emergency) adj3 (dispatcher or personnel))).tw. (14573)
15 or/11-14 (14908)
16 10 and 15 (325)

CINAHL

**S18**	**S10 AND S16**<br>**Limiters - Published Date: 20200701-20211231**	**705**
S17	S10 AND S16	742
S16	S11 OR S12 OR S13 OR S14 OR S15	101,647

S15	TI ((Military or paramilitary or armed force* or veterans or armed service* or servicewomen or servicemen or air-personnel or defense force* or defence force* or service personnel or navy or air force or sailor* or soldier* or infantryman or Civil-defense or Troops or coast guard or submariner* or active duty or enlisted personnel or reserve personnel or police officer* or RCMP or firefighter* or firem* or EMT or EMTs or EMS or (Emergency Medical adj2 (personnel or technician*)) or paramedic* or (public safety adj3 (professional* or official* or personnel*)) or "first responder*" or "search and rescue" or ambulance or ((law enforcement or corrections or correctional) adj (worker* or staff or personnel or officer*)) or security guard* or security personnel or sheriff* or (border adj3 (agent* or personnel or security)) or emergency manager* or (("911" or emergency) adj3 (dispatcher or personnel)))) OR AB ((Military or paramilitary or armed force* or veterans or armed service* or servicewomen or servicemen or air-personnel or defense force* or defence force* or service personnel or navy or air force or sailor* or soldier* or infantryman or Civil-defense or Troops or coast guard or submariner* or active duty or enlisted personnel or reserve personnel or police officer* or RCMP or firefighter* or firem* or EMT or EMTs or EMS or (Emergency Medical adj2 (personnel or technician*)) or paramedic* or (public safety adj3 (professional* or official* or personnel*)) or "first responder*" or "search and rescue" or ambulance or ((law enforcement or corrections or correctional) adj (worker* or staff or personnel or officer*)) or security guard* or security personnel or sheriff* or (border adj3 (agent* or personnel or security)) or emergency manager* or (("911" or emergency) adj3 (dispatcher or personnel))))	77,666
S14	(MH "Emergency Medical Technicians")	12,883
S13	(MH "Police") OR (MH "Firefighters")	15,897
S12	(MH "Veterans") OR (MH "Vietnam Veterans") OR (MH "Veterans Health Services")	19,167
S11	(MH "Military Services") OR (MH "Military Deployment") OR (MH "Military Dependents") OR (MH "Military Family")	3,664
S10	S1 OR S2 OR S3 OR S4 OR S5 OR S6 OR S7 OR S8 OR S9	41,874
S9	TI ((para-professional* or paraprofessional*)) OR AB ((para-professional* or paraprofessional*))	759
S8	TI ((mutual support or mutual help or support group*)) OR AB ((mutual support or mutual help or support group*))	7,100
S7	(MH "Support Groups")	11,044
S6	TI (community adj2 network*) OR AB (community adj2 network*)	0
S5	(MH "Community Networks")	2,599
S4	TI ((buddy or buddies)) OR AB ((buddy or buddies))	769

S3	TI ((peer led or peer based or peer deliver)) OR AB ((peer led or peer based or peer deliver))	1,200
S2	TI ((peer* N3 (support or group* or mentor* or lead* or coach* or counsel* or service* or specialist* or outreach or network* or volunteer* or education*))) OR AB ((peer N3 (support or group* or mentor* or lead* or coach* or counsel* or service* or specialist* or outreach or network* or volunteer* or education*)))	13,107
S1	(MH "Peer Group") OR (MH "Peer Counseling")	15,725

Web of Science

# 10	**1090**	#8 AND #7 **Refined by: PUBLICATION YEARS:** (2021 OR 2020 OR 2010 OR 2000 OR 2019 OR 2009 OR 2018 OR 2008 OR 2017 OR 2007 OR 2016 OR 2006 OR 2015 OR 2005 OR 2014 OR 2004 OR 2013 OR 2003 OR 2012 OR 2002 OR 2011 OR 2001)
# 9	1155	#8 AND #7
# 8	400373	TS=(Military or paramilitary or "armed force*" or veterans or "armed service*" or servicewomen or servicemen or air-personnel or "defense force"* or "defence force*" or "service personnel " or army or navy or "air force" or sailor* or soldier* or infantryman or "Civil-defense" or Troops or "coast guard" or submariner* or "active duty" or "enlisted personnel" or "reserve personnel" or "police officer"* or RCMP or firefighter* or firem* or EMT or EMTs or EMS or (Emergency Medical NEAR/2 (personnel or technician*)) or paramedic* or (public safety NEAR/3 (professional* or official* or personnel*)) or "first responder*" or "search and rescue" or ambulance or (("law enforcement" or corrections or correctional) NEAR (worker* or staff or personnel or officer*)) or "security guard*" or "security personnel" or sheriff* or (border NEAR/3 (agent* or personnel or security)) or "emergency manager*" or (("911" or emergency) NEAR/3 (dispatcher or personnel)))
# 7	72850	#6 OR #5 OR #4 OR #3 OR #2 OR #1
# 6	2063	TS= (para-professional* or paraprofessional*)
# 5	1917	TS=(buddy or buddies)
# 4	13544	TS=(community NEAR/2 network*)
# 3	12755	TS=("support group*" or "mutual support" or "mutual help")

# 2	5742	TS=("peer led" or "peer based" or "peer deliver*" or "peer enhanced") OR AK=peer support
# 1	42504	TS=(peer NEAR/3 (support or group* or mentor* or lead* or coach* or counsel* or service* or specialist* or outreach or network* or volunteer* or education*))
